# Regarding the rights and duties of Clinical Laboratory Geneticists in genetic healthcare systems; results of a survey in over 50 countries

**DOI:** 10.1038/s41431-019-0379-4

**Published:** 2019-03-28

**Authors:** Thomas Liehr, Isabel M. Carreira, Zsofia Balogh, Elena Dominguez Garrido, Irmgard Verdorfer, Domenico A. Coviello, Lina Florentin, Hans Scheffer, Martina Rincic, Heather E. Williams

**Affiliations:** 1 Jena University Hospital, Friedrich Schiller University, Institute of Human Genetics, Jena, Germany; 2 Laboratory of Cytogenetics and Genomics, Faculty of Medicine, University of Coimbra, Coimbra, Portugal; 3 Research Centre for Environment, Genetics and Oncobiology, Coimbra, Portugal; 4 Experimental and Translational Pathology Platform, Inserm US23/CNRS UMS3655, AMMICa, Gustave Roussy Cancer Campus, Villejuif, France; 5 Fundación Rioja Salud, Unit of Molecular Diagnostic, Logroño-La Rioja, Spain; 6 Division of Human Genetics, Medical University of Innsbruck, Innsbruck, Austria; 7 Laboratory of Human Genetics, E.O. Ospedali Galliera, Genova, Italy; 8 Alfalab, Molecular Biology and Cytogenetics Center, Leto Maternity Hospital, Athens, Greece; 9 Department of Human Genetics, Radboud University Medical Center, Nijmegen, The Netherlands; 10 Croatian Institute of Brain Research, Zagreb, Croatia; 11 Viapath at King’s College Hospital, Haematological Malignancy Diagnostic Centre, Cytogenetics Laboratory, London, UK

**Keywords:** Clinical genetics, Medical genetics

## Abstract

Specialists of human genetic diagnostics can be divided into four groups: Medical Geneticists (MDG), Genetic Nurses and/or Counsellors (GN/GC), Clinical Laboratory Geneticists (CLG) and Laboratory Genetics Technicians (LGT). While the first two groups are in direct patient contact, the work of the latter two, of equal importance for patient care, are often hidden as they work behind the scenes. Herein the first study on the rights and duties of CLGs is presented. We present the results of a survey performed in 35 European and 18 non-European countries with 100 participating specialists. A national CLG title is available in 60% of European countries, and in 77% of the surveyed European countries a CLG can be the main responsible head of the laboratory performing human genetic tests. However, in only 20% of European countries is a lab-report valid with only a CLGs’ signature - even though the report is almost always formulated by the CLG, and an interpretation of the obtained results in a clinical context by the CLG is expected in nearly 90% of European countries. Interestingly, CLGs see patients in 30% of European countries, and are also regularly involved in student education. Overall, the CLG profession includes numerous duties, which are quite similar in all regions of the world. Strikingly, the CLG’s rights and responsibilities of leading a lab, or signing a report are regulated differently according to country specific regulations. Overall, the CLG is a well-recognized profession worldwide and often working within a multidisciplinary team of human genetic diagnostics professionals.

## Introduction

Human genetic diagnostics is an emerging field, as knowledge of underlying causes of inherited and acquired diseases continues to grow exponentially [1]. This explosion has led to the utilization of genetic testing by a wide range of healthcare providers and patients, as direct to consumer tests have allowed in home testing. As previously outlined [2], these developments are principally positive for patient care. Conversely, the quality of diagnostic tests offered by non-human genetic trained specialists has become a subject of great concern [2]. Thus, the European Board of Medical Genetics (EBMG) together with the European Society of Human Genetics (ESHG) and the EuroGentest, formerly a Network of Excellence [3], now an integrated committee in the ESHG, are providing tools for improving the quality of genetic testing in Europe. This aim is to be achieved via: (i) certification of specialists in human genetic diagnostics, and (ii) appropriate genetic education for healthcare professionals. The work carried out by this united group reveals the need for concerted efforts to provide professional education to a range of health professionals who play a role in the provision of genetic testing [2, 4–6]. EBMG recommends that a human genetic diagnostics multidisciplinary team is necessary, and is exemplified by the inclusion of four groups of professional experts [7]: Medical Geneticists (MDG), Genetic Nurses & Genetics Counsellors (GN/GC), Clinical Laboratory Geneticists (CLG) and Laboratory Genetics Technicians (LGT). While the working conditions of MDGs and GNs/GCs have been studied in detail [8–10], no such data is available concerning rights and duties of CLGs or LGTs in different countries. Herein we address this gap for CLGs through a survey completed with over 50 countries worldwide and 100 participants.

## Materials and methods

The survey (Supplementary data 1) included three blocks: (1) two questions concerning anonymity and consent to participate in the study, (2) six questions on demographic background of the participant, and  (3) 10 questions on rights and duties of CLGs in the corresponding country. This survey was sent from August to December 2017 to 222 CLGs in 88 countries. As Greece developed state recognition of a national CLG title in May 2018, this was included in the study, accordingly. The final evaluation was completed through assessment of questions included in the survey, with responses grouped by reported country (Supplementary data 1):

1. Is there a national CLG-title in your country

If so:

- Who provides the title?
- Is the title state recognized as a profession?

2. Can a CLG be head of the laboratory performing human genetic tests?
3. Is the report only valid with signature of an MD?
4. Who receives the final report?
5. Is the final report written by the CLG him-/herself?
6. Can further tests be suggested by the CLG?
7. Is there any result interpretation in report?
8. Does the CLG see and/or counsel patients?
9. Do CLGs teach at universities?

If so:

- Is there a difference if they work in private or in university laboratory?

Answers for survey questions (see S﻿upplem﻿entary data 1: e.g., 7a, 7 h, 7j) led to inconclusive answers and/or responses indicated the question was outside of the knowledge of the interviewed participants (S﻿upplem﻿entary data 1: e.g., 4 and 9) and thus were excluded in the final evaluation.

## Results

Evaluable survey replies (Supplementary data 1) were returned from 53/88 countries (60%; 35 European and 18 non-European countries) and 100/222 CLGs (45%). There were 1 and 2 replies per country for 28 and 10 countries, respectively, and 3 and 4 replies per country for 8 and 7 countries, respectively (Tab. 1).

**Fig. 1:**
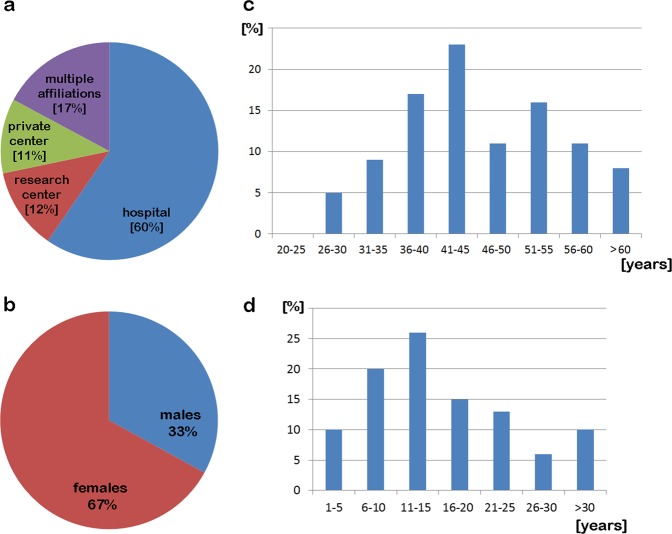
Statistics on participating 100 CLGs or equivalent specialists interviewed in the present study; type of center witnesses are working within (**a**), gender (**b**), age-distribution (**c**) and years of work experience (**d**) are given

The demographic data of the CLGs are presented by age-characteristics in Fig. 1. The majority of respondents worked at a hospital (60%), 11 and 12% in private and research centers, respectively, and 17% worked at multiple locations in parallel (Fig. 1a). The respondent ratio of females to males was 2:1 (Fig. 1b); the age distribution and work experience of participants are shown in Fig. 1c, d.

The responses of CLGs in the 53 countries were as follows:

1. A national CLG-title is available in 60% of the 35 European countries surveyed, and only 28% of the 18 non-European (Fig. 2). National CLG titles were provided by national human genetic societies or by government. Exclusive to Australia and USA, CLG titles issued by national human genetic societies are state recognized as a legal professional title. In all other countries, only certificates issued by the state are recognized legally.

**Fig. 2:**
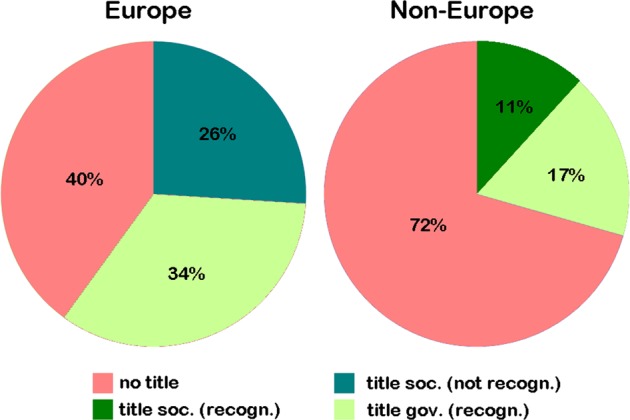
Availability of CLG title in 35 European and 17 non-European countries is depicted; also indicated is whether the national CLG title is state recognized as a legal profession. Abbreviations: *gov* government, *recogn.* state recognized, *soc* society
2. A CLG can be the main responsible head of the laboratory performing human genetic tests in 73% of the countries (in 10% there are some exceptions; see footnotes Tab. 1), and in only 27% of the countries must an MD be head of the laboratory. Within Europe this accounts for 23% of the countries, i.e., in 77% of the European countries, a CLG may be head of a human genetic diagnostic laboratory. This statistic was reflected not surprisingly by the response of a Dutch colleague to question 5b regarding MD involvement: ‘Certainly a CLG can be responsible head of a lab without an MD at his/ her side. How could an MD be responsible for a lab?'.
3. A human genetic report is only valid with a signature of an MD in 1/3 of the 53 countries. In Europe, the signature of an MD is required for validity in only 1/5 of the countries. Interestingly, in some countries instead of an MD, LGTs or a second CLG co-sign the report. The validity of a report must be considered in the context of reimbursement by health insurance companies for services rendered. In USA, a report with MD signature is reimbursed at a higher rate by insurance companies, with reports of lower reimbursement for an identical report with CLG signature only. Conversely, e.g., in Netherlands and Switzerland it is logically concluded that the CLG signature is the reason for reimbursement of laboratory diagnostics given these tests are completed under their aegis.
4. In greater than 95% of the countries, the final report goes to the MD who requisitioned the diagnostic test. In fewer than 5% of countries, the patient or family receives the report. In addition, in some countries GNs/GCs or midwifes are recipients of reports.
5. In all European countries excluding Turkey, and only 5 non-European countries is the final report written by the CLG.
6. Further tests can be suggested by a CLG in all European countries excluding Russia and Turkey, and 6 non-European countries. In nearly 90% of the countries, the expertise of CLGs is well-recognized and reflected in their ability to requisition additional tests to optimize patient care.
7. Result interpretation in a clinical context within the report is expected in 31/35 European and 12/18 non-European countries.
8. CLGs see and/or counsel patients alone or with MDs in nearly 30% of European and 22% of non-European countries. In some countries participation in MD-lead genetic counselling is obligatory during CLG education.
9. CLGs teach at universities in all European countries, excluding Denmark, and in all non-European countries excluding Iraq. In addition, teaching activities are dependent on the employer (private or public).

## Discussion

The present survey is, to the best of our knowledge, the first performed on the working conditions of CLGs. The responses were collected from 100 CLGs (Table 1). Their age distribution and length of work experience support the conclusions of this study as the answers were given by experienced CLGs. Interestingly, the gender distribution of CLGs participating in this survey is similar to previously recognized findings of the CLG profession [2]. The recruitment of participants was mainly based on personal contacts of Thomas Liehr. Where possible, for each country at least two CLGs were contacted; in addition, for each country, the representatives as listed on https://www.ebmg.eu/666.0.html as national contacts for ErCLG related questions were included, and for non-European countries, either registered affiliated ErCLGs and/or heads of corresponding human genetic societies were contacted. However, for 28 countries only one contacted representative participated. In general, the more people who replied for each country, the more comprehensive the country specific data became for data evaluation. This can be contributed to the fact that a CLG knows best their working environment, and may hold additional information regarding working conditions of one or more laboratories within the country. Differences from general rules are expected between countries, given local specificities are abundant. Local specificities were evident in this survey when contradictory answers were given by participants in the same country, and when multiple respondents reported detailed comments on their country. However, conflicting questions and their corresponding responses were thus excluded from final evaluation. The results of this survey have been made freely available on EBMG webpage (https://www.ebmg.eu/910.0.html). New data on further countries and/or corrections on information described herein are welcome and will be uploaded when sent to Thomas.Liehr@med.uni-jena.de.

**Table 1:** Results of the survey in 53 countries and 100 CLGs

Country name	Number of witnesses	National Title (provided by)	National Title (state recognized)	CLG maybe head of a lab?	Report is only valid with MD sign.?	Report is received by…	Report is written by CLG?	Further tests can be sugge- sted by CLG?	Is there result interpretation in report?	CLGs see / counsel patients?	CLGs teach at university when working at university/ in private institution?
1. Albania	1	N (n.a.)	n.a.	Y	N	P	Y	Y	Y	Y	Y/N
2. Armenia	3	N (n.a.)	n.a.	Y	N	MD / P	Y	Y	Y	N	Y/Y
3. Australia	1	Y (genet. soc.)	Y	Y	N	MD / P	Y	Y	Y	N	Y/Y
4. Austria	2	Y (genet. soc.)	N^b^	Y	N	MD	Y	Y	Y	N	Y/N
5. Belarus	2	N (n.a.)	n.a.	N	Y	MD / P	Y	Y	Y	N	Y/Y
6. Belgium	1	Y (genet. soc.)	N	Y	Y	MD	Y	Y	Y	N	Y/N
7. Bosnia & Herzegovina	1	N (n.a.)	n.a.	N	Y	MD / P	Y	Y	Y	Y	Y/Y
8. Brazil	2	N (n.a.)	n.a.	Y	N	MD / P	Y	Y	N	N	Y/N
9. Canada	3	Y (gouv.)	Y	Y^c^	N	MD	Y	Y	Y	N	Y/N
10. China	1	N (n.a.)	n.a.	N	Y	MD / P	N	Y	N^m^	Y	Y/N
11. Croatia	4	N (n.a.)	n.a.	Y^d^	N	MD / P	Y	Y	Y	Y (rarely)	Y/Y
12. Cuba	1	N (n.a.)	n.a.	Y	Y	n.a.p.	N	N	n.a.p.	N	n.a.p.
13. Cyprus	1	N (n.a.)	n.a.	Y	N	MD	Y	Y	Y	Y	Y/Y
14. Czech Republic	1	Y (genet. soc.)	N	Y	Y	MD / P	Y	Y	Y	Y	Y/Y
15. Denmark	1	Y (genet. soc.)	N	Y	N	MD	Y	Y	Y	N	Y/Y
16. Ecuador	3	N (n.a.)	n.a.	N	Y	MD / P	Y	N	N	N	Y/Y
17. Finland	4	Y (gouv.)	Y	Y^e^	N^e^	MD	Y	Y	Y	N	Y/Y
18. France	2	Y (gouv.)	Y	N	Y	MD / P	Y	Y	Y	N	Y/Y
19. Germany	4	Y (genet. soc.)	N	N	Y	MD	Y	Y	Y	N^n^	Y/Y
20. Greece	4	Y (gouv.)	Y	Y	N	MD / P	Y	Y	Y	Y	Y/Y
21. Hungary	4	Y (gouv.)	Y	Y	N	MD	Y	Y	Y	N	Y/Y
22. India	1	N (n.a.)	n.a.	Y	N	MD / P	Y	Y	Y	N	Y/Y
23. Iran	1	N (n.a.)	n.a.	Y	N	MD / P	Y	Y	Y	Y (rarely)	Y/Y
24. Iraq	1	N (n.a.)	n.a.	N	Y	MD	N	N	Y	N	N/N
25. Ireland	2	N (n.a.)	n.a.	N	N^g^	MD	Y	Y	Y	Y	Y/Y
26. Israel	3	Y (gouv.)	Y (partly)	Y	N	MD	Y	Y	N	N	Y/N
27. Italy	4	Y (gouv.)	Y	Y	N	MD / P	Y	Y	Y	Y^o^	Y/Y
28. Latvia	1	Y (gouv.)	Y	Y	N	MD	Y	Y	Y	N	Y/Y
29. Macedonia	1	N (n.a.)	n.a.	N	Y	MD	Y	Y	N	N	Y/N
30. Montenegro	1	N (n.a.)	n.a.	N	Y	MD	Y	Y	N	N	Y/N
31. Morocco	1	N (n.a.)	n.a.	N	Y	MD	N	Y	N	N	Y/Y
32. Netherlands	3	Y (genet. soc.)	N	Y^f^	N	MD / MW	Y	Y	Y	N	Y/n.a.
33. Norway	1	Y (genet. soc.)	N	Y	n.a.p.	MD	Y^l^	Y	Y	N	Y/n.a.
34. Pakistan	1	N (n.a.)	n.a.	Y	N^h^	P	Y	Y	Y	Y	Y/Y
35. Poland	4	Y (gouv.)	Y	Y	N^i^	MD / GC~GN	Y	Y	Y	N	Y/N
36. Portugal	1	Y (gouv.)^a^	Y^**1**^	Y	N	MD	Y	Y	Y	N	Y/Y
37. Romania	2	N (n.a.)	n.a.	Y	N^h^	MD	Y	Y	Y	N	Y/N
38. Russia	2	Y (gouv.)	Y	Y^e^	N^h^	MD / P	Y	N	N	N	Y/Y
39. Serbia	3	N (n.a.)	n.a.	Y	N^h^	MD / P	Y	Y	Y	N^p^	Y/Y
40. Slovakia	1	Y (genet. soc.)	N	Y	N	MD / P	Y	Y	Y	Y (rarely)	Y/Y
41. Slovenia	2	Y (gouv.)	Y	Y	N	MD / P	Y	Y	Y	N^p^	Y/n.a.
42. South Africa	1	N (n.a.)	n.a.	Y	N	MD	Y	Y	Y	N	Y/Y
43. Spain	2	N (n.a.)	n.a.	Y	N	MD	Y	Y	Y	Y	Y/Y
44. Sweden	3	Y (genet. soc.)	N	Y	N	MD / MW / P	Y	Y	Y	N	Y/N
45. Switzerland	1	Y (gouv.)	Y	Y	N^i^	MD	Y	Y	Y	N^n^	Y/n.a.
46. Syria	1	N (n.a.)	n.a.	Y	N	MD / P	Y	Y	Y	Y	n.a.p.
47. Tunisia	1	N (n.a.)	n.a.	N	Y^j^	MD	Y	N	Y	N	Y/Y
48. Turkey	3	N (n.a.)	n.a.	N	Y	MD / P	N	N	N	N	Y/n.a.
49. UK	1	Y (gouv.)	Y	Y	N	MD	Y	Y	Y	N	Y/n.a.
50. Ukraine	2	N (n.a.)	n.a.	Y	N^i^	MD / P	Y	Y	Y	N	Y/Y
51. Uruguay	1	N (n.a.)	n.a.	N	Y	MD	Y	Y	Y	N	Y/n.a.
52. USA	1	Y (genet. soc.)	Y	Y	N^k^	MD / GC~GN	Y	Y	Y	N	Y/Y
53. Yemen	1	N (n.a.)	n.a.	Y	N	MD / P	Y	N	N	N	Y/Y

^a^at present not provided

^b^CLG profession mentioned in one law (see https://www.jusline.at/gesetz/gtg), but CLGs are not state recognized

^c^except for Quebec

^d^exception biochemical genetic reporting

^e^only in private, not in hospital

^f^labs are part of Clinical Genetics departments

^g^but there must be a second signature

^h^second signature by person doing lab work

^i^second signature by person doing lab work or colleague

^j^report exclusively with MD and no CLG signature

^k^report without MD signature leads to lower reimbursement of insurance

^l^in parts prepared by technicians

^m^maybe some in tumorcytogenetic cases

^n^have to be present in genetic counselling several times during CLG training

^o^only for pre-test counselling and informed consent explanation

^**16**^ can be present within a team;

Abbreviations: GC~GN = genetic counsellor or nurse; MD = medical doctor; MW = midwife; N = no; n.a. = not applicable; n.a.p. = no answer provided; P = patient; Y = yes

Herein, for the first time, the differences in working conditions of CLG in non-European and European countries are reported. In general, the profession is more widely recognized in Europe, North-America, and Australia, rather than other parts of the world. Accordingly, working conditions of European, Canadian, US-American and Australian CLGs are more regulated. Their duties and influence on diagnostics are well-defined given their nationally (Tab. 1) or on European level [2] certifiable expertise. An EU wide recognition of the CLG title is more than overdue, and is now possible following the rules of EU Directive 2005/36/EC—policy developments (https://www.eshg.org/fileadmin/eshg/EBMG/CLG/Direttiva_36_EN.pdf) and the “Proposal for modernising the Professional Qualifications Directive” = EU Directive 2013/55/EU (https://www.eshg.org/fileadmin/eshg/EBMG/CLG/Direttiva_55_EN.pdf), as at present (i.e. March 2018) Finland, France, Greece, Italy, Hungary, Latvia, Poland, Slovenia and UK have national state recognized CLG titles. Efforts to (re-)achive this kind of national recognition, are currently underway in Portugal, Spain, Cyprus, and Denmark. The EBMG is in preparation of starting this EU CLG recognition process (https://www.ebmg.eu/668.0.html).

As the majority of the surveyed countries not only allow a CLG to be head of the laboratory performing human genetic tests, but also to sign responsibly without an MD demonstrates the already high acceptance rate of the CLG profession. Examples from Norway, Poland, Pakistan, Romania, Russia, Serbia, Switzerland, and Ukraine suggest that even more responsibility in reporting can be successfully shared with LGTs, too.

Undoubtedly, reports of human genetic laboratory diagnostics primarily go via the requesting MD to the patient or family nearly everywhere in the world. However, it is noteworthy that GNs/GCs or midwifes (valid for result of non-invasive prenatal diagnostic tests = NIPT [11]) can also be the recipients and disseminators of reports in some countries. The MDs and/or GNs/GCs are also responsible for the genetic counselling and consulting of patients given genetic testing results [8–10]. As such, as a minimum, during their education, a CLG should experience patient counselling and become familiar with the dissemination of genetic testing information to patients, which will ensure all parties understand how clinical queries are solved in part by human genetic laboratory diagnostics [12]. In addition, nearly everywhere the report is competently written by the CLG and (if applicable) further tests are recommended, and further interpretation provided. Finally, the CLG profession is warmly welcomed and integrated into student education in nearly all surveyed countries.

## Conclusions

CLG is a well-recognized profession worldwide. CLGs have clearly defined rights and duties within a multidisciplinary team composed of four professions: MDGs, GNs/GCs, CLGs and LGTs, working diligently to provide patients reliable diagnoses with a focus on ‘precision medicine’ for all [13]. In Australia, North-America, and the majority of European countries, the vital role of CLG in patient care is reflected highly given state recognition of the profession. Thus, the European Union should wait no longer, and move quickly forward with recognition of the CLG profession as soon as possible, as previously recommended by OECD (https://www.eshg.org/fileadmin/www.eshg.org/documents/QAGuidelineseng.pdf).

## Supplementary information

Supplementary data 1
