# Supplementary material for: Regarding the rights and duties of Clinical Laboratory Geneticists in genetic healthcare systems; results of a survey in over 50 countries
Source: Eur J Hum Genet. 2019 Mar 28;27(8):1168–74. doi: 10.1038/s41431-019-0379-4 (PMC6777624; doi:10.1038/s41431-019-0379-4)
Supplement: Supplementary file 1 — Supplementary data 1 [file 41431_2019_379_MOESM1_ESM.docx]

Questionnaire for (Er)CLGs
= European registered Clinical Laboratory Geneticists

STUDY NAME Feedback on the duties and rights of (Er)CLGs in different countries
AIM This project is aimed at exploring the duties and rights of (Er)CLGs in different countries.

Please return asap to [Thomas.Liehr@med.uni-jena.de](mailto:Thomas.Liehr@med.uni-jena.de)

Form may be filled by computer – you may mark answers or delete non-relevant answers

INFORMED CONSENT

Thank you for agreeing to complete this survey. You were invited to participate in this study because you are listed on <https://www.eshg.org/666.0.html> as a national representative and contact for the “European Board of Medical Genetics Professional Branch Board for Clinical Laboratory Geneticists”, who undertakes this study; also you may be contacted as a registered Clinical Laboratory Geneticist. Participation in this study is completely voluntary. Your answers to all questions are confidential. The answers will help us duties and rights of (Er)CLGs in different countries and may also be bases for a publication. Thank you for your help!

I understand my answers will be anonymous

- - Yes
  - No

I consent to be involved in this study

- - Yes
  - No

**Demographics**

a. Gender

- - Female
  - Male

b. Age

- - 20-25 years
  - 26-30 years
  - 31-35 years
  - 36-40 years
  - 41-45 years
  - 46-50 years
  - 51-55 years
  - 56-60 years
  - over 60 years

c. Country: _________________________________

d. Place of work (tick all that apply)

- - Hospital
  - Research centre
  - Private institution
  - Laboratory
  - Other: ________________________

e. What is your job title / current position ? __________________________________

h. How many years of experience do you have in Clinical Laboratory Genetics?

- - less than 1 year
  - 1 year
  - 2 years
  - 3 years
  - 4 years
  - 5 years
  - 6-10 years
  - 11-15 years
  - 16-20 years
  - 21-25 years
  - 26-30 years
  - over than 30 years

**Specific Questions**

1. I fill this form for _____________ *(insert country please)*
2. We have a national CLG title – I refer here in the following to the title:
   ______________________________________ *(insert title please or state that you have none)*
3. If you have national CLG title in your country: is it recognized by your state/ government / medical insurance companies? Or is it only provided by e.g. a genetic society? If latter is the case, which efforts are undertaken to get state/ government recognition?
4. Is there an official directive/law as to how and under which conditions a clinical genetics lab can function? Please provide all possible documents that you may know about.
5. Can an (Er)CLG be the head of the lab in your country (in state hospitals and similar)?
   - Yes
   - No
6. If so: Which role has an (Er)CLG in making the choice of a genetic study to be performed, when material was sent to the lab? Is the test already ‘ordered’ by the providing person, or is there a multidisciplinary team (included (Er)CLG), who makes the decision, or does this the head of the lab, or do (Er)CLGs call the provider of the probe to specify what needs to be done?
7. If so: Can an (Er)CLG be responsible head of a lab without an MD at his/ her side?
8. If not: Is it obligatory by law for the lab director to be a medical doctor? If yes – which specialization is necessary for the MD?
9. If so: Do (Er)CLGs have a follow up system for the patients studied in the lab?
10. Do (Er)CLGs teach at university …
    1. if they work at university?
    2. if they work in private company?
11. Do (Er)CLGs write reports themselves?
    - Yes
    - No
12. If so: Do (Er)CLGs have regular and close dialogue with the referring Physician (Oncologist, Clinician, Pathologist…)?
13. If so: Who is the recipient of the report (patient, another lab, an MD; if an MD is it a medical geneticist)?
14. If so: In case of tumor diagnostics do (Er)CLGs comment on possible diagnostics / treatment possibilities / prognostics?
15. If so: In case of constitutional diagnostics do (Er)CLGs only report results, or do they also provide interpretation? If latter is valid, is it like a or maybe instead of a genetic counselling letter?
16. If so: Is the report valid with (Er)CLGs sole signature?
17. If so: Who cosigns the reports beside (Er)CLGs?
18. If so: Is the report valid without signature of an MD?
19. If so: Do (Er)CLGs act as contact person for report relevant questions/demands/explanations to the referring physician, or is that to be done by an MD?
20. If so: Can (Er)CLGs recommend additional testing if necessary?
21. If so: Do (Er)CLGs receive the relevant and sufficient clinical data from referring physician/ person?
22. Do (Er)CLGs see patients?
    - Yes
    - No
23. If so: Do (Er)CLGs counsel them, or is that to be done by an MD or a Genetic Counselor?
24. If valid: Does your national CLG-providing organization organize meetings for ongoing education?
25. Additional comments for this study:

Thanks a lot for your support!!
